# Analysis of Draft Genome Sequence of* Pseudomonas* sp. QTF5 Reveals Its Benzoic Acid Degradation Ability and Heavy Metal Tolerance

**DOI:** 10.1155/2017/4565960

**Published:** 2017-11-15

**Authors:** Yang Li, Yi Ren, Nan Jiang

**Affiliations:** ^1^Tianjin Institute of Industrial Biotechnology, Chinese Academy of Sciences, Tianjin, China; ^2^Shanghai Majorbio Bio-Pharm Biotechnology Co., Ltd, Shanghai, China; ^3^Institute of Applied Ecology, Chinese Academy of Sciences, Shenyang, China

## Abstract

*Pseudomonas* sp. QTF5 was isolated from the continuous permafrost near the bitumen layers in the Qiangtang basin of Qinghai-Tibetan Plateau in China (5,111 m above sea level). It is psychrotolerant and highly and widely tolerant to heavy metals and has the ability to metabolize benzoic acid and salicylic acid. To gain insight into the genetic basis for its adaptation, we performed whole genome sequencing and analyzed the resistant genes and metabolic pathways. Based on 120 published and annotated genomes representing 31 species in the genus* Pseudomonas*, in silico genomic DNA-DNA hybridization (<54%) and average nucleotide identity calculation (<94%) revealed that QTF5 is closest to* Pseudomonas lini* and should be classified into a novel species. This study provides the genetic basis to identify the genes linked to its specific mechanisms for adaptation to extreme environment and application of this microorganism in environmental conservation.

## 1. Introduction

The Qiangtang basin is a region of continuous permafrost located in the Qinghai-Tibetan Plateau of China. It is an extreme environment with low temperatures, high UV radiation, and few nutrients. It is the largest petroleum-bearing basin in the plateau [1]. Our previous study [2] investigated the microbial composition and diversity of this area and found that Proteobacteria was the second largest phylum (following Actinobacteria), ranging from 18.7 to 20.43%. The dominant class was Gammaproteobacteria, to which the genus* Pseudomonas* belongs.

*Pseudomonas* is aerobic and metabolically diverse, allowing it to occupy a wide range of niches [3]. Many species in the* Pseudomonas* genus are known for their resistance and survival in the presence of several organic and inorganic pollutants [4], including heavy metals [5], cyanide [6], normal hydrocarbons, and aromatic compounds [7]. Furthermore, certain species of* Pseudomonas* have been used for biocontrol [8] or bioremediation [9].

*Pseudomonas* sp. QTF5 was isolated from the soil sample near bitumen, which is the impermeable rock formation of petroleum [10]. It was chosen for whole genome sequencing because it is psychrotolerant and highly and widely tolerant to heavy metals and has the ability to metabolize benzoic acid and salicylic acid. Here, we present a summary of the classification and characteristics of QTF5, together with a description of the draft genome sequence and annotation. It is identified based on the phylogenetic placement of its 16S rDNA sequence as well as pairwise digital DNA-DNA hybridization (dDDH) values and average nucleotide identity (ANI).

## 2. Materials and Methods

### 2.1. Strain Isolation and Selection

Frozen soil was collected from the continuous permafrost beside the bitumen at 5,111 m above sea level in the Qiangtang basin [2]. Bacteria were originally isolated on PYGV agar medium at 15°C.* Pseudomonas* sp. strain QTF5 was selected on the basis of its heavy metal resistance and benzoic acid degrading ability using a modified method described by Jebeli et al. [12]. Cells of strain QTF5 were examined in a HT7700 Transmission electron microscope (Hitachi, Tokyo, Japan) and a SU8010 scanning electron microscope (Hitachi, Tokyo, Japan). This strain was deposited in China General Microbiological Culture Collection Center (CGMCC) under accession number 1.15161.

### 2.2. Whole Genome Sequencing, Assembly, and Annotation

Cells of strain QTF5 were harvested from LB broth following overnight incubation at 30°C with shaking at 180 rpm. Genomic DNA was extracted using Genomic DNA Purification Kit (Fermentas, USA) according to the manufacturer's instruction. The genome of strain QTF5 was sequenced using the Illumina HiSeq 2000 and Miseq platforms. Two libraries of 400 bp and 600 bp insert size were generated and sequenced by 2 × 100 bp and 2 × 300 bp paired-end runs. A total of 2.4 Gb high quality data (15,971,017 reads) were assembled with the SOAP de novo assembler (v2.04) [13], providing approximately 410-fold coverage. The coding sequences (CDSs) were predicted using Glimmer 3.0 [14] and their function was annotated through comparisons with databases of NR [15], COG [16], and KEGG [17]. The quality score of assembled sequences, rRNAs, tRNAs, and essential genes was calculated according to the algorithm described by Land et al. [18]. The quality score of genome sequences was assigned based on a combination of contigs and nonstandard bases. The rRNA score was calculated based on the length of predicted 5S, 16S, and 23S rRNAs. The tRNA score was based on predicted tRNAs, at least one of which codes for each of the 20 standard amino acids. A neighbor joining phylogenetic tree was constructed based on 16s rDNA sequences of QTF5 and other published representative strains of 19 species in genus* Pseudomonas*, with* Azomonas* as an outgroup. The tree uses the Jukes-Cantor corrected distance model to construct a distance matrix. Bootstrap values above 30%, based on 1,000 replications, are shown at the branching points. GenBank accession number for each strain is shown in parenthesis.

### 2.3. Genome Wide Comparative Analysis

In order to further resolve the taxonomy of the new isolate, comparative genomic analysis was used to compare QTF5 with 120 genomes that represent 31 species across the genus* Pseudomonas* (Table S1 in Supplementary Material available online at https://doi.org/10.1155/2017/4565960). ANI was computed from protein coding genes of the genomes with the Jspecies program using default parameters [19]. dDDH values and their confidence intervals were determined using Genome-to-Genome Distance Calculator (GGDC 2.1 http://ggdc.dsmz.de) under recommended settings [20].

### 2.4. Assessment of Heavy Metals Resistance and Benzoic Acid Degradation

The level of resistance was determined for heavy metals in their salt forms according to a Maximum Inhibitory Concentration (MIC) method described previously [12]. The salts used were as follows: ZnSO_4_ (Zn), Pb(NO_3_)_2_ (Pb), CuSO_4_ (Cu), MnCl_2_ (Mn), CoCl_2_ (Co), NiSO_4_ (Ni), K_2_Cr_2_O_7_ (Cr), and HgCl_2_ (Hg). To investigate the degradation ability of benzoic acid and its analogs, cultures were harvested after 4 h to obtain log phage cells. Washed cells were suspended in basic inorganic medium and then diluted to a concentration of 5.0 × 10^6^ cells/ml. 200 *μ*l of the cell suspension (i.e., 10^6^ cells) was added to three individual wells of a microtiter plate containing 100 *μ*l of water, sodium benzoate (final concentration: 0.03%) or salicylic acid (final concentration: 0.18%), respectively. Individual wells in the same plate containing 200 *μ*l basic inorganic medium and 100 *μ*l one were used as the controls. Each treatment was repeated 4 times. The plates were incubated at 30°C with shaking for 3 days and then centrifuged at 4000 rpm for 10 mins. The absorption spectrum of supernatant in each well was measured in an automated spectrophotometer SpectraMax® M5 (Molecular Devices, LLC, Sunnyvale, California) [21].

### 2.5. Nucleotide Sequence Accession Number

The genome sequenced as part of this study has been deposited at GenBank under accession number AZRW00000000. The version described here represents an improved assembly and is the second version AZRW00000000.2.

## 3. Results and Discussion

### 3.1. General Feature of* Pseudomonas* sp. Strain QTF5

**Figure 1:**
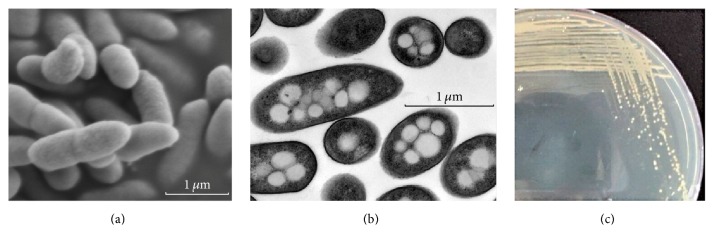
Characteristic images of* Pseudomonas* sp. QTF5. (a) Scanning electron microscopy image; (b) transmission electron microscopy image; (c) image of colonies on agar plate.

Strain QTF5 was isolated from frozen soil cultivated on PYGV medium at 15°C. It is Gram-negative, nonmotile, and rod-shaped measuring 0.5 *μ*m in diameter and 1.5 *μ*m in length (Figure 1). When incubated with fresh nutrient medium, QTF5 forms organized lumps on the agar surface, leading to the growth of dry opaque, pale orange, concentrically ringed colonies within 2 days. It could grow at a broad temperature range from 10 to 42°C, with an optimum at 30°C, at pH 5.6–8.0 (optimum at pH 6.6–7.0).

### 3.2. Physiological Characteristics of Strain QTF5

Tolerance of strain QTF5 to a wide range of heavy metals was assessed using MIC tests under optimal growth condition. Strain QTF5 is resistant to with MICs up to 32.0 mM (Zn), 12.8 mM (Pb), 6.4 mM (Cu), 4.0 mM (Mn), 3.2 mM (Co), 3.2 mM (Ni), 1.6 mM (Cr), and 0.01 mM (Hg), respectively. The highest MICs of other heavy metal resistant* Pseudomonas *strains described previously are lower than QTF5, or some strains are only resistant to one or several of these heavy metals [22–25]. For example,* P. aeruginosa* J007 and PAO1 are only resistant to three heavy metals. Specifically, the MICs of J007 were determined to be of 3 mM (Cu), 2 mM (Cd), and 6 mM (Zn), and those of PAO1 were 2 mM (Cu), 0.125 mM (Pb), and 8 mM (Zn). Although* P. aeruginosa* CCTCC AB93066 is resistant to a wider range of heavy metals, the MICs of most heavy metals are much lower than QTF5, accounting for 0.97 mM (Pb), 1.57 mM (Cu), 0.83 mM (Co), and 1.70 mM (Ni). Compared with those reported strains, QTF5 has a much broader resistant spectrum and relatively higher tolerant level.

**Figure 2:**
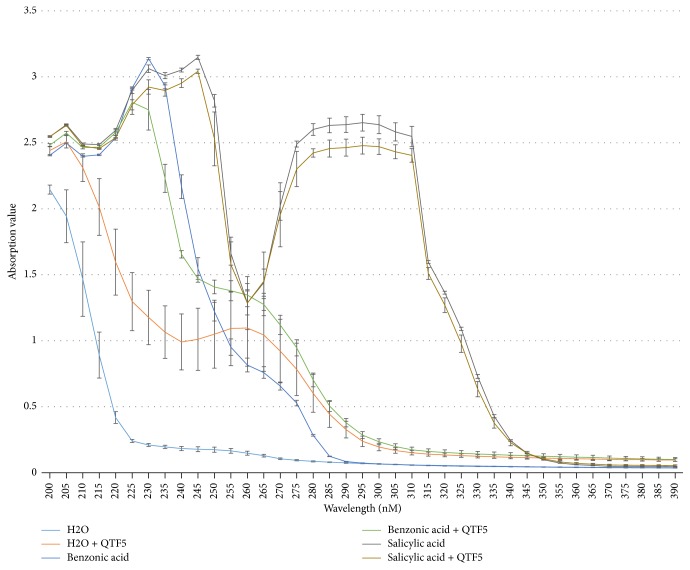
UV spectrum demonstrates the degradation of benzoic acid and salicylic acid by strain QTF5. The absorption value for each wavelength is the mean value of four replicated trials. The error bars indicate standard deviation.

Moreover, strain QTF5 is able to utilize benzoic acid as the sole carbon and energy source for growth in basic medium. The degradation of benzoic acid involve several oxidation steps and the products cause increase of peak height in absorption spectra [26]. QTF5 can significantly change the absorption spectra (Figure 2), especially in the characteristic wavelength of benzoic acid (230 nM) and salicylic acid (235 nM, 305 nM) as reported previously [26–29]. Specifically, after adding the strain QTF5, OD_230_ significantly decreased from 3.14 to 2.75 in the wells with benzoic acid (*p* < 0.05), and OD_235_ significantly decreased from 3.01 to 2.90 and OD_305_ from 2.58 to 2.43 in the wells with salicylic acid (*p* < 0.05).

### 3.3. Genome Assembly and Gene Prediction

The draft genome consists of 90 scaffolds, which are composed of 101 contigs. The N50 length is 129 kb and the largest contig approximately 293 kb. The quality score of assembled sequences, rRNAs, tRNAs, and essential genes are 0.87, 0.9, 0.9, and 0.99, respectively. The final quality score of the draft genome is 0.929. The genome is composed of a circular chromosome without any extrachromosomal elements. The genome size was approximately 6,019,946 bp with a G + C content of 58.71% (Table 1). A total of 5,589 genes were predicted, 5,524 of which are protein coding genes, and 65 are RNA genes. 4,450 (79.74%) of the protein coding genes were assigned to a putative function with the remaining annotated as hypothetical proteins or proteins of unknown functions.

### 3.4. Functional Annotation and Analysis

According to the KO assignment and KEGG pathway mapping, 2,849 (51.57%) protein coding genes of strain QTF5 could be assigned to 112 metabolic pathways. Metabolic pathways consist of the most abundant gene set (*n* = 778, 14.08% of total protein coding genes), followed by biosynthesis of secondary metabolites (347, 6.28%), microbial metabolism in diverse environments (256, 4.63%), and two-component system (170, 3.08%). Using COG function assignment, 4,848 of protein coding genes could be classified into 22 COG categories. The properties and the statistics of the genome are summarized in Table 2. The most abundant category of metabolism, information storage and processing, and cellular processes and signaling are related to amino acid transport and metabolism (488, 8.83%), transcription (414, 7.49%), and signal transduction mechanisms (371, 6.72%).

### 3.5. Taxonomical Classification

**Figure 3:**
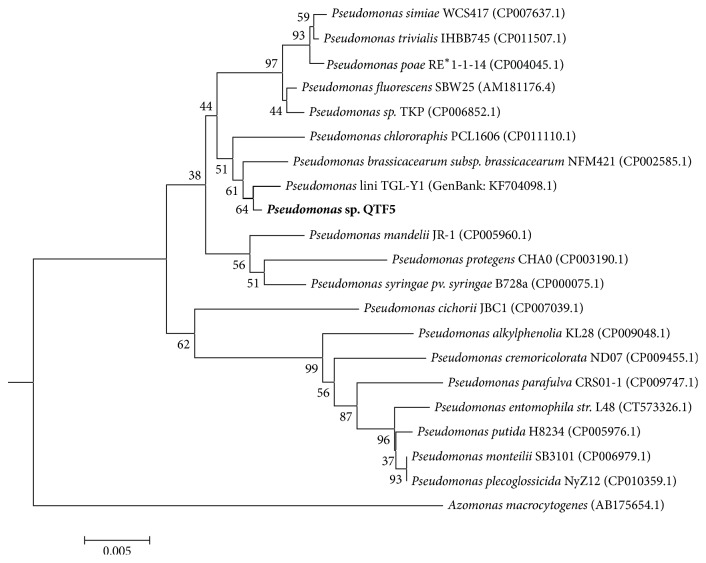
A neighbor joining phylogenetic tree of 16S rDNA gene highlights the position of* Pseudomonas* sp. QTF5 relative to other strains within the genus* Pseudomonas*. GenBank accession number for each strain is shown in parenthesis. The tree uses the Jukes-Cantor corrected distance model to construct a distance matrix. Bootstrap values are shown at the branching points.* Azomonas macrocytogenes* [11] was used as an outgroup.

The phylogenetic position of genus* Pseudomonas* is in the Pseudomonadaceae, a very diverse family within the order Pseudomonadales, the phylum Proteobacteria. The closest related genera are* Azomonas* and* Azotobacter* [30, 31]. A phylogenetic tree of 16S rDNA sequences reveals that* P. lini* [32] TGL-Y1 (GenBank: KF704098.1) and* Pseudomonas brassicacearum* subsp.* brassicacearum* [33] NFM421 (GenBank: CP002585.1) are the closest phylogenetic neighbors of QTF5 (Figure 3). The former stain was isolated, like QTF5, from Qinghai-Tibet Plateau and described as a crude oil degrading bacterium. In silico GGDC and ANI showed 54.1% and 94.01% identity to the closest relative,* P. lini*, respectively. The detailed results are shown in Table S1.

Genomic taxonomy can be studied through various parameters including average nucleotide identity and Genome BLAST Distance Phylogeny [20, 34]. Depending on the methods used for ANI calculation or the nature of bacterial genome sequences, 95 or 96.5% ANI value [35, 36] corresponds to the classical 70% DNA-DNA relatedness cutoff value [37] for strains of the same species. The highest dDDH and ANI value between QTF5 and reference strain is far lower than the cutoff value, confirming that QTF5 belongs to a novel species within genus* Pseudomonas*.

### 3.6. Identification of Heavy Metal Resistance and Benzoic Acid Degradation Genes

**Figure 4:**
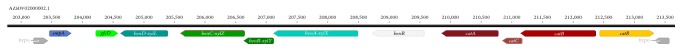
Genetic structure of benzoic acid degradation gene clusters,* ben* operon and* cat* operon, was detected in scaffold AZRW02000002.1 (scheme). The location and polarity of genes are shown with arrows. Hypo: hypothetical protein;* smpA*: membrane protein;* yfcD*: NUDIX hydrolase;* benD-xylL*: 1,6-dihydroxycyclohexa-2,4-diene-1-carboxylate dehydrogenase;* benC-xylZ*: benzene 1,2-dioxygenase reductase component;* benB-xylY*: benzene 1,2-dioxygenase subunit beta;* benA-xylX*: benzene 1,2-dioxygenase subunit alpha;* benR*:* ben* operon regulatory protein;* catA*: catechol 1,2-dioxygenase;* catC*: muconolactone delta-isomerase;* catB*: muconate cycloisomerase;* catR*:* cat* operon regulatory protein.

All the genes involved in iron (III) transport system, iron complex transport system, and high-affinity zinc uptake system are present in this genome. Two copies of lead, cadmium, zinc, and mercury transporting ATPases, two copper resistance gene clusters, a heavy metal efflux system, nine heavy metal efflux and resistance genes, and two heavy metal responsive transcriptional regulators were identified, which may explain the high tolerance of this strain. Two gene clusters responsible for benzoate degradation,* ben* operon (204,627–209,577, AZRW02000002.1) and* cat* operon (209,854–213,318, AZRW02000002.1), were found on the genome (Figure 4), indicating the ability to metabolize benzoic acid. Seven cold-shock proteins were found in this genome including* cspA*,* cspC,* and* cspD*. The recombination related genes* recA*,* recF*,* recG*,* recN*,* recO*,* recQ*,* radA*,* radC*, and single-stranded-DNA-specific exonuclease gene* recJ*, which play a critical role in DNA damage repair, were also found. The cold-shock, recombination repair of damaged DNA, cyclic hydrocarbon degradation, and heavy metal efflux proteins are significant for this bacterium to survive the extreme environment in Tibetan plateau near bitumen, which has low temperature, high UV radiation, high heavy metal concentration, and low nutrition.

## 4. Conclusions

Genome analysis of a novel* Pseudomonas* sp. strain QTF5 revealed a high degree of consistency between genotypes and phenotypes, especially in heavy metal resistance, benzoic acid degradation, and psychrotolerant characteristic. Genome sequence of QTF5 provides insight into better understanding of the molecular mechanism of the genus* Pseudomonas* in extreme environment adaptation.

Oilfield wasteland or petroleum-contaminated soil is always associated with high concentration and broad spectrum of heavy metals and aromatic compounds, which are difficult to remove and degrade [38, 39]. Heavy metal resistant bacteria can be efficient bioremediators of metals and may provide an alternative or additional method to conventional methods to remove them. Species of* Pseudomonas *have been proved to be highly effective in biosorption of metals [40] and QTF5 may be also used for enhanced remediation of contaminated environment. In addition, this strain has been shown to degrade benzoic acid, which is a major pollutant in petroleum-contaminated areas [39]. Strain QTF5 could potentially be used for biotechnological exploitation for perspective petroleum reservoir and bioremediation of environmental pollution.

## Supplementary Material

Comparative analysis with 120 published genomes representing 31 species in the genus *Pseudomonas*.

## Figures and Tables

**Table 1:** Genome statistics.

Attribute	Value	% of total^*∗*^
Genome size (bp)	6,019,946	100
Coding region (bp)	5,167,653	85.84
G + C content (bp)	3,534,328	58.71
RNA genes	65	1.163
Protein-coding genes	5,524	85.69
Genes with function prediction	4,405	79.74
Genes assigned to COGs	4,848	87.76
Genes assigned to TIGRfam domains	1,561	28.26
Genes assigned to Pfam domains	2,546	46.09
Genes with signal peptides	521	9.432
Genes with transmembrane helices	1213	21.96

^*∗*^The total is based on either the size of the genome in base pairs or the total number of protein coding genes in the annotated genome.

**Table 2:** Number of genes associated with the 25 general COG functional categories.

Code	Value	% age^*∗*^	Description
A	1	0.02	RNA processing and modification
B	3	0.05	Chromatin structure and dynamics
C	278	5.03	Energy production and conversion
D	41	0.74	Cell cycle control, cell division, chromosome partitioning
E	488	8.83	Amino acid transport and metabolism
F	91	1.65	Nucleotide transport and metabolism
G	254	4.60	Carbohydrate transport and metabolism
H	197	3.57	Coenzyme transport and metabolism
I	189	3.42	Lipid transport and metabolism
J	182	3.29	Translation, ribosomal structure and biogenesis
K	414	7.49	Transcription
L	170	3.08	Replication, recombination and repair
M	289	5.23	Cell wall/membrane/envelope biogenesis
N	128	2.32	Cell motility
O	189	3.42	Posttranslational modification, protein turnover, chaperones
P	225	4.07	Inorganic ion transport and metabolism
Q	127	2.30	Secondary metabolites biosynthesis, transport and catabolism
R	568	10.28	General function prediction only
S	433	7.84	Function unknown
T	371	6.72	Signal transduction mechanisms
U	145	2.62	Intracellular trafficking, secretion, and vesicular transport
V	65	1.18	Defense mechanisms
W	0	—	Extracellular structures
Y	0	—	Nuclear structure
Z	0	—	Cytoskeleton
—	676	12.24	Not in COGs

^*∗*^The total is based on the total number of protein coding genes in the annotated genome.
